# The Association between* Helicobacter pylori* Infection and Chronic Hepatitis C: A Meta-Analysis and Trial Sequential Analysis

**DOI:** 10.1155/2016/8780695

**Published:** 2016-01-24

**Authors:** Juan Wang, Wen-Ting Li, Yi-Xiang Zheng, Shu-Shan Zhao, Ning Li, Yan Huang, Rong-Rong Zhou, Ze-Bing Huang, Xue-Gong Fan

**Affiliations:** ^1^Department of Infectious Diseases, Xiangya Hospital, Central South University, Changsha 410008, China; ^2^Key Laboratory of Viral Hepatitis, Hunan, China; ^3^Department of Orthopedics, Xiangya Hospital, Central South University, Changsha 410008, China; ^4^Department of Blood Transfusion, Xiangya Hospital, Central South University, Changsha 410008, China

## Abstract

*Purpose*.* Helicobacter pylori* is a common gastric disease-inducing pathogen. Although an increasing number of recent studies have shown that* H. pylori* is a risk factor for liver disease, the potential association between* H. pylori* infection and chronic hepatitis C still remains controversial. The aim of our meta-analysis was to evaluate a potential association between* H. pylori *infection and chronic hepatitis C.* Methods*. We searched the PubMed, Embase, CNKI, Web of Science, and the Cochrane Central Register of Controlled Trials (CENTRAL) databases between January 1, 1994, and May 1, 2015.* Results*. This study included a total of 1449 patients with chronic hepatitis C and 2377 control cases. The prevalence of* H. pylori* was significantly higher in patients with chronic hepatitis C than in those without chronic hepatitis C. The pooled odds ratio was 2.93. In a subgroup analysis, the odds ratios were 4.48 for hepatitis C virus- (HCV-) related cirrhosis and 5.45 for hepatocellular carcinoma.* Conclusion*. Our study found a strong association between* H. pylori* and chronic hepatitis C, particularly during the HCV progression stage; thus, we recommend active screening for* H. pylori* in patients with chronic hepatitis C.

## 1. Introduction

*Helicobacter pylori* (*H. pylori*) is a Gram-negative spiral-shaped bacteria that colonizes the gastric mucosa and can induce chronic gastritis, gastric ulcers, and gastric malignancy [1]. Worldwide, approximately 50% of the population is infected with* H. pylori* [2]. Both developing and developed countries have a high incidence of* H. pylori* infection [3]. The rate of* H. pylori* infection correlates significantly with socioeconomic conditions and the age at infection. In addition, all* H. pylori*-infected individuals subsequently develop gastritis [4].

Hepatitis C virus (HCV) was first recognized in 1989. To date, nearly 180 million people worldwide have been infected with HCV. If not well controlled, chronic hepatitis C (CHC) can progress into cirrhosis and ultimately into hepatocellular carcinoma (HCC) [5, 6], which might lead to higher patient mortality rates [7]. To date, no effective medicines have been developed to prevent the progression of CHC to cirrhosis and/or HCC because the mechanism by which cirrhosis or HCC occurs is not fully understood. However, the hepatitis viral load, genotype, and infection duration are known to play important roles in the development and progression of HCC [8].

Increasing evidence suggests that* H. pylori* may be a risk factor for the development of cirrhosis and HCC in patients with CHC [8–10]; however, some researchers have suggested that* H. pylori* might not contribute to the mechanism of HCV-related HCC [11, 12]. The relationship between* H. pylori* and CHC remains controversial, and it is unclear whether* H. pylori* is associated with CHC and the progression of HCV-related cirrhosis and HCC. In this study, we aimed to confirm the associations between* H. pylori* and CHC and to further assess the relationship of* H. pylori* with HCV-related cirrhosis and HCC through a meta-analysis. Therefore, the results of this study might propose clinical medicine approaches for patients with CHC who might develop cirrhosis or HCC.

## 2. Materials and Methods

### 2.1. Eligibility Criteria

We searched PubMed, Embase, Web of Science, China National Knowledge Infrastructure (CNKI), and Cochrane Central Register of Controlled Trials (CENTRAL) databases from January 1, 1994, to May 1, 2015. We used keywords or subject headings to search for “hepatitis C” and “helicobacter pylori or helicobacter species.” There were no language restrictions. We also screened bibliographies of selected original studies, review articles, and relevant conference abstracts.

Citations were merged in Endnote version X7 (Thomson Reuters, New York, NY, USA) to facilitate management. Two reviewers (J. Wang and Y.-X. Zheng) independently applied the inclusion criteria to all retrieved studies. Disagreements between reviewers were resolved by consensus. For each eligible study, the following criteria were set and reviewed independently by the reviewers: (1) Eligible study designs were randomized controlled trials (RCTs), case-control studies, or cross-sectional studies comparing* H. pylori*-related morbidity between patients with CHC and a control group with nonhepatic disease. (2) Concrete numbers of cases and controls and a* H. pylori* positivity rate were included. (3) The study groups exhibited definite HCV positivity, whereas the control groups were HCV-negative. (4)* H. pylori* was detected in all study groups through serological or PCR testing. (5) Studies presenting information exclusively about patients undergoing liver transplantation, viral hepatitis (e.g., hepatitis A, B, or E virus), human immunodeficiency virus, acute HCV, autoimmune liver disease, nonalcoholic fatty liver disease (NAFLD), and other types of hepatitis were excluded.

### 2.2. Data Extraction and Quality Assessment

For each eligible study, the following data elements were selected: first author, publication year, country,* H. pylori* detection method, the number of cases and controls, the prevalence of* H. pylori* infection in cases and controls, and numbers of HCC, cirrhosis, and noncirrhosis cases among patients with CHC. Twelve studies, all case-control studies, were included after consensus was achieved between the two reviewers. The observational study quality was assessed using the Newcastle-Ottawa Scale (NOS) [13, 14]. This scale scores studies across three categories: selection (four stars), comparability of study groups (two stars), and assessment of outcome/exposure (three stars). The star rating system is used to indicate the quality of a study, and a maximum score of nine stars is possible; the studies included herein were graded on an ordinal star-scoring scale with higher scores indicating a higher quality.

## 3. Statistical Analysis

RevMan 5.2 (Nordic Cochrane Centre, Cochrane Collaboration, Copenhagen, Denmark) was used to perform the meta-analysis. We used TSA, version 0.9 (Copenhagen Trial Unit, Copenhagen, Denmark), to assess the required information size. Data were combined when appropriate and displayed in forest plots. Risk ratios (RRs) were used to assess risk in cohort studies; odds ratios (ORs) were provided for case-control studies and were regarded as approximate RRs in this meta-analysis. Study heterogeneity was tested using the chi-square test and *I* ^2^ statistics. If significant heterogeneity (chi-square test, *P* < 0.10 and *I* ^2^ > 50%) was found, the random-effect model was used for the analysis; if the heterogeneity was considered to be insignificant (chi-square test, *P* ≥ 0.10 and *I* ^2^ < 50%), on the other hand, the fixed-effect model was used. Studies with substantial heterogeneity (*I* ^2^ > 50%) were considered unsuitable for the meta-analysis. Subgroup and sensitivity analyses were used to discover the source of heterogeneity. Confounding factors were segregated for further analysis.

## 4. Result

**Figure 1:**
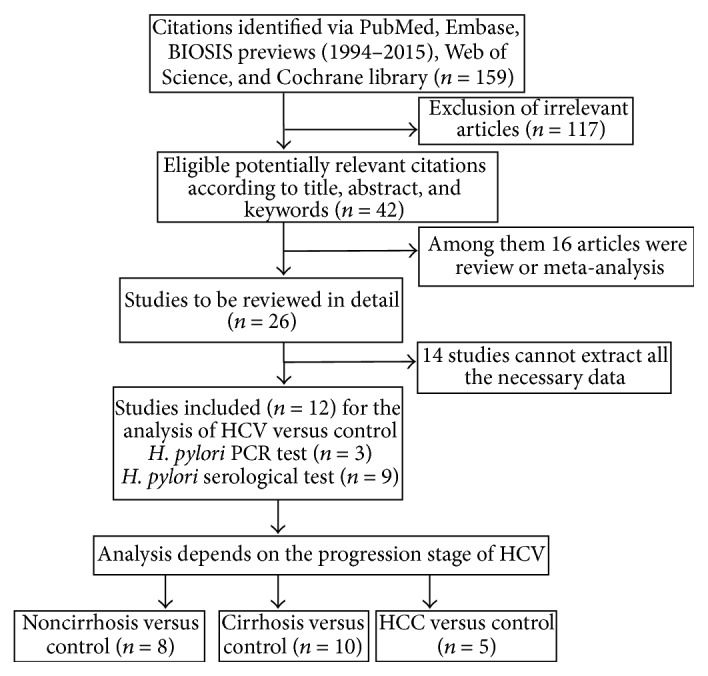
Flow diagram of study selection and analysis. HCV: hepatitis C virus; HCC: hepatocellular carcinoma.

Twelve case-control studies were included from among a total of 159 studies identified through online database searches. Most ineligible studies were excluded on the basis of information in the title or abstract. The selection and analysis process is shown in a flow diagram in Figure 1. The characteristics of included studies [15–26] are shown in Table 1.

### 4.1. *H. pylori* Positivity Rates of the HCV and Control Groups

**Figure 2:**
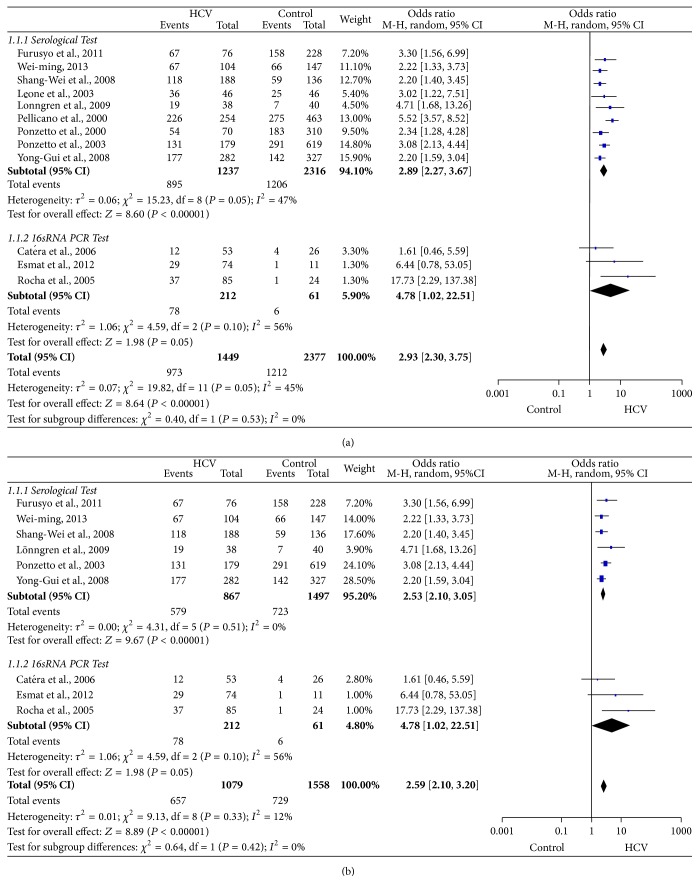


In the meta-analysis of 12 studies (Figure 2(a)), a pooled OR of 2.93 (95% confidence interval [2.30, 3.75]; *P* = 0.05) was determined, indicating a 2.93-fold higher* H. pylori* positive rate among patients with CHC than among healthy controls. Whereas all 12 studies exhibited significant heterogeneity (*I* ^2^ equals 45%) in the total analysis, we divided the included studies into two subgroups based on the* H. pylori* detection method. We found that the subtotal pooled OR of the PCR test subgroup was obviously higher than that of the serological test subgroup (4.78 versus 2.89) and the *I* ^2^ values of both subgroups nearly 50% (56% and 47%, resp.). The major source of interstudy heterogeneity might, therefore, have originated from other confounding factors. As shown in the study characteristics (Table 1), three articles [19, 20, 26] limited the HCV groups to patients with cirrhosis or HCC. Therefore, disease progression might be an influencing factor. We conducted a sensitivity analysis using a gradual elimination process and found that if we excluded these three studies [19, 20, 26], the heterogeneity decreased to the lowest value shown in Figure 2(b) (total group *I* ^2^ = 12%, serological group *I* ^2^ = 0%). According to the above information, patients with CHC appear to have a nearly 2.59-fold higher* H. pylori* positivity rate when compared to healthy controls. However, disease stage was the main factor influencing the* H. pylori* positive rate among patients with CHC. Therefore, further analysis of the extracted data according to HCV progression stage is needed.

### 4.2. Subgroup Analysis Based on HCV Progression Stage

**Figure 3:**
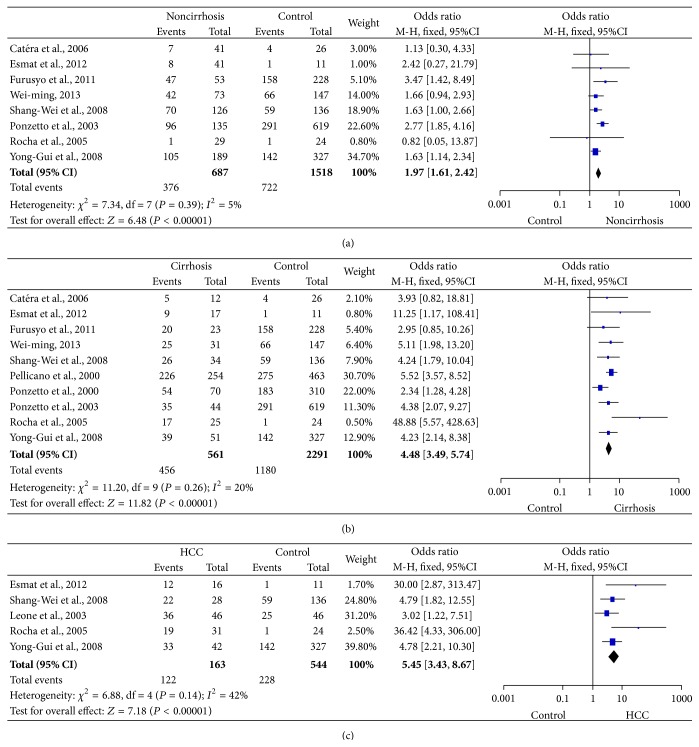
Forest plots of a meta-analysis depending on HCV progression stage. HCC: hepatocellular carcinoma; M-H: Mantel-Haenszel.

To attenuate the influence of the HCV disease stage on the meta-analysis, we further generalized the extracted data from included studies to a noncirrhosis group, cirrhosis group, and HCC group, all of which were compared with the control group. Forest plots of all meta-analysis are shown in Figure 3. The* H. pylori* positivity rate among patients with CHC without cirrhosis was 1.97-fold higher than that of the healthy controls (pooled OR = 1.97, 95% CI: 1.61–2.42; *P* = 0.39). The* H. pylori* positivity rate among patients with CHC having cirrhosis was 4.48-fold higher (pooled OR = 4.48, 95% CI: 3.49–5.74; *P* = 0.26) than that of the control group, and the comparable rate among patients with HCV-related HCC was 5.45-fold higher (pooled OR = 5.45, 95% CI [3.43, 8.67]; *P* = 0.14). All *I* ^2^ values for the above analyses were <50% (*I* ^2^ = 5%, 20%, and 42%, resp.), indicating that the studies exhibited low heterogeneity with respect to each other in the three meta-analyses. The extracted data were suitable for analysis, and the results were reliable because of the deep analysis enabled by disease stage ranking.

### 4.3. Trial Sequential Analysis

**Figure 4:**
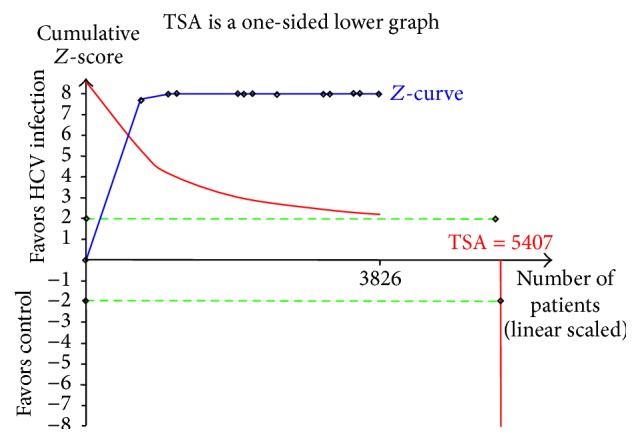
Trial sequence analysis of* H. pylori* infection in patients with HCV versus controls.

Trial sequential analysis (TSA) combines conventional meta-analysis methodology with repeating significance testing methods applied to accumulating data in clinical trials. TSA uses cumulative *Z*-curves to assess relationships with conventional significance boundaries (*Z* = ±1.96), the required information size, and the trial sequential monitoring boundaries. In our trial sequential analysis, the type I error risk was set at *α* = 0.05 with a power of 0.80. Relative risk reduction (RRR) was defined by the average incidence of included studies at 10%. Our determined required information size was 5407. In fact, the included number was 3826. From Figure 4, we concluded that although the *Z*-curve did not reach the required information size (RIS), it crossed the trial sequential monitoring boundary and conventional boundary, which show the result was true-positive.

### 4.4. Evaluation of Publication Bias

**Figure 5:**
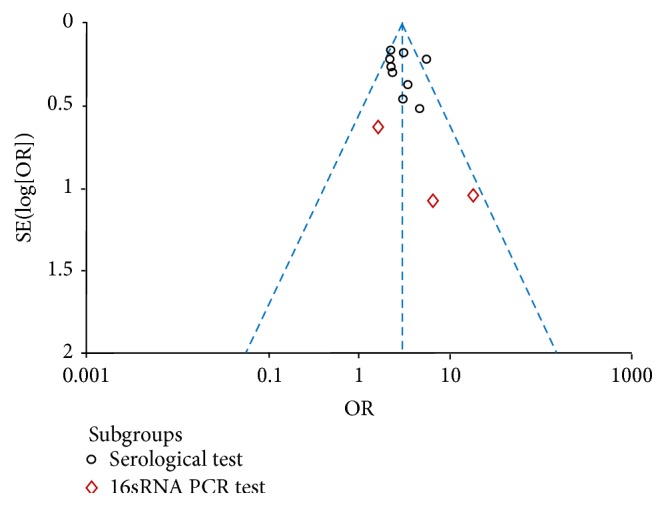
Funnel plot to explore publication bias. The graphical funnel plot of the 12 published studies appears to be symmetrical.

A funnel plot is used to assess publication bias for outcomes >10. We included 12 studies in this meta-analysis. This evaluation is shown in Figure 5. The graph appears as a symmetrical inverted funnel, indicating a very low risk of publication bias.

## 5. Discussion 

*H. pylori* infection is a major global health issue. In addition, the World Health Organization has recognized* H. pylori* as a group 1 carcinogen contributing to gastric cancer since 1994 [27]. Furthermore, numerous lines of evidence indicate an association between* H. pylori* and liver disease, particularly CHC [28]. Although an association between* H. pylori* infection and CHC has been reported, it remains controversial. Several studies have shown a strong correlation between* H. pylori* infection and CHC and HCV-related disease. In contrast, other studies maintained that* H. pylori* infection does not correlate with CHC development. We conducted this study to assess the association between the* H. pylori* infection rate and CHC.

The result of our meta-analysis indicates a higher prevalence of* H. pylori* among patients with CHC relative to controls. In other words,* H. pylori* infection is strongly associated with CHC. In addition, TSA provided firm evidence to support our results. Otherwise, the rate of* H. pylori* infection would continue to increase with CHC progression. This could implicate* H. pylori* as a risk factor for CHC progression. The role of* H. pylori* in CHC and CHC development were supported by considerable evidence. Sakr et al. also reported that the histopathological changes were more severe in patients with* H. pylori*positive HCV than in patients with HCV alone [29]. Furthermore, Umemura et al. demonstrated that the eradication of* H. pylori* could increase sustained virological responses in patients with CHC [30]. Moreover, many studies conducted* in vitro* demonstrated a cytopathic effect of* H. pylori* on hepatocytes [31, 32]. Therefore, our results are consistent with the above viewpoints.

Moreover, the subgroup analysis demonstrated a 4.48-fold higher* H. pylori *incidence rate (95% CI: 3.49–5.74) among patients with HCV-related cirrhosis relative to the control group, which strongly indicates a correlation between late-stage cirrhosis and* H. pylori* infection. However, two [16, 19] of the 10 studies reported no association between HCV and Child-Pugh cirrhosis staging, whereas the remaining studies did not discuss this issue. However, all included articles strongly indicated a correlation between late-stage cirrhosis and* H. pylori* infection. Furthermore, El-Masry et al. reported an increasing incidence of* H. pylori* as the Child-Pugh class increased [33]. Given the limited number of articles, future studies should address the relationship between* H. pylori* infection and the Child-Pugh class in patients with HCV-related cirrhosis.

Finally, the OR was 5.45-fold higher in the HCC group than the control group. This finding obviously indicates a strong link between* H. pylori* and HCC. Many clinical observational studies have reported the detection of* H. pylori* in abnormal liver tissues, particularly cancerous tissues [28]. A meta-analysis conducted by Xuan et al. demonstrated a positive association between* H. pylori* and HCC. The patients with HCC in that study had diverse etiologies. Only two of the articles included in that study were related to HCV-positive HCC [34]. Therefore, the observations of Xuan and colleagues agree with our study findings.

Our study had some potential limitations. Firstly, the final results of this meta-analysis were greatly affected by the limitations of the included publications. Although we attempted to identify all related studies, it is possible that some escaped our attention. Furthermore, some studies with negative results might not have been published, which would cause unavoidable publication bias. In addition, all articles identified in our search were in the English and Chinese languages, which may have caused language bias. Regarding the control group source, most included studies used blood donors as controls, although some studies used hospital samples. This might have also generated bias.

Secondly, the* H. pylori* detection methods differed among the included studies, a factor that might have led to different positivity rates. Some of the included studies detected and diagnosed* H. pylori* infection via the serology method, whereas others used PCR-based methods. Usually, the serology method is less sensitive and specific [35]. Meanwhile, both* H. pylori *and* H. hepaticus* belong to the* Helicobacter* genus [36].* H. pylori* might share genetic sequences with other* Helicobacter* species such as* H. hepaticus*. Therefore, diagnosis of* H. pylori *infection via PCR alone is not sufficient.

Thirdly, nearly all included articles were case-control studies. The quality of this type of study is lower than that of randomized clinical trials. In addition, both age and socioeconomic status have been considered high-risk factors for* H. pylori* infection [27]. El-Masry et al. reported that* H. pylori*-infected individuals older than 40 years had a high risk of severe HCV-related disease [33]. However, Wei-ming only included older people in their study, whereas the studies conducted by Yong-Gui et al., Shang-Wei et al., and Pellicano et al. included subjects aged 20–89 years; none of the other included studies mentioned the subjects' ages. The results would have been better if those authors had balanced the incidence of* H. pylori* infection with respect to ages. Moreover, the patients in our study resided in different countries, including China, Italy, France, Egypt, Sweden, and Japan. Therefore, the* H. pylori* incidence might have varied with respect to socioeconomic differences. This might have caused the misestimating of the odds ratios.

In conclusion, our meta-analysis demonstrated a positive association between* H. pylori* infection and CHC. In particular, it also revealed strong correlations of* H. pylori* infection with HCV-related cirrhosis and HCV-related HCC. We suggest the importance of* H. pylori* screening of patients with chronic hepatitis, particularly those with HCV-related cirrhosis and/or HCC. Obviously, given the limitations of the included studies, the role of* H. pylori* as a risk factor in the progression of CHC remains unclear. We recommend conducting additional randomized controlled trials and prospective studies to clarify the effect of this pathogen on CHC development.

## Figures and Tables

**Table 1:** Characteristics of included studies.

Study	Country	NOS stars	*H. pylori* detection method	*H. pylori* positivity/total subjects		*H. pylori* positivity in different HCV progression stages		
				Control	HCV	Noncirrhosis	Cirrhosis	HCC
Catéra et al. 2006 [15]	France	7^*∗*^	16sRNA PCR test	4/26	12/53	7/41	5/12	NR
Esmat et al. 2012 [16]	Egypt	6^*∗*^	16sRNA PCR test	1/11	29/74	8/41	9/17	12/16
Furusyo et al. 2011 [17]	Japan	8^*∗*^	Serological test	158/228	67/76	47/53	20/23	NR
Lönngren et al. 2009 [18]	Sweden	7^*∗*^	Serological test	7/40	19/38	NR	NR	NR
Pellicano et al. 2000 [19]	Italy	6^*∗*^	Serological test	275/463	226/254	NR	226/254	NR
Ponzetto et al. 2000 [20]	Italy	8^*∗*^	Serological test	183/310	54/70	NR	54/70	NR
Ponzetto et al. 2003 [21]	Italy	7^*∗*^	Serological test	291/619	131/179	96/135	35/44	NR
Rocha et al. 2005 [22]	France	6^*∗*^	16sRNA PCR test	1/24	37/85	1/29	17/25	19/31
Shang-Wei et al. 2008 [23]	China	8^*∗*^	Serological test	59/136	118/188	70/126	26/34	22/28
Wei-ming 2013 [24]	China	6^*∗*^	Serological test	66/147	67/104	42/73	25/31	NR
Yong-Gui et al. 2008 [25]	China	7^*∗*^	Serological test	142/327	177/282	105/189	39/51	33/42
Leone et al. 2003 [26]	Italy	7^*∗*^	Serological test	25/46	36/46	NR	NR	36/46

NR: not reported; HCV: hepatitis C virus; HCC: hepatocellular carcinoma; NOS: Newcastle-Ottawa Scale.
